# Screening for Stereopsis Using an Eye-Tracking Glasses-Free Display in Adults: A Pilot Study

**DOI:** 10.3389/fmed.2021.814908

**Published:** 2022-01-17

**Authors:** Fang Liu, Jing Zhao, Tian Han, Yang Shen, Meng Li, Jingrong Liu, Dong Yang, Yong Fang, Li Yan, Xingtao Zhou

**Affiliations:** ^1^Department of Ophthalmology and Optometry, Eye, Ear, Nose, and Throat Hospital, Fudan University, Shanghai, China; ^2^National Health Commission Key Laboratory of Myopia (Fudan University), Key Laboratory of Myopia, Chinese Academy of Medical Sciences, Shanghai, China; ^3^Shanghai Research Center of Ophthalmology and Optometry, Shanghai, China; ^4^Shanghai EVIS Technology Co., Ltd., Shanghai, China; ^5^National Engineering Research Center for Healthcare Devices, Guangzhou, China

**Keywords:** stereopsis screening, glasses-free display, eye-tracking, random-dot, feasibility

## Abstract

**Purpose:**

To explore the feasibility and repeatability of a novel glasses-free display combined with random-dot stimulus and eye-tracking technology for screening stereopsis in adults.

**Methods:**

A total of 74 patients aged 18–44 years were recruited in this study (male: female, 32:42), including 33 patients with high myopia [≤ -6.0 diopters (D)] and 41 patients with moderate-to-low myopia (>-6.0 D). Stereopsis was measured using glasses-free, polarized, and Titmus stereotests. All patients completed a visual fatigue questionnaire after the polarized stereotest and glasses-free test. Kendall's W and Cohen's Kappa tests were used to evaluate repeatability and consistency of the glasses-free stereotest.

**Results:**

The stereotest results using the glasses-free monitor showed strong repeatability in the three consecutive tests (*W* = 0.968, *P* < 0.01) and good consistency with the polarized stereotest and Titmus test results (vs. polarization: Kappa = 0.910, *P* < 0.001; vs. Titmus: Kappa = 0.493, *P* < 0.001). Stereopsis levels of the high myopia group were significantly poorer than those of the moderate-to-low myopia group in three stereotest monitors (all *P* < 0.05). There was no significant difference in visual fatigue level between the polarized and the glasses-free display test (*P* = 0.72). Compared with the polarized test, 56.76% of patients preferred the glasses-free display and found it more comfortable, 20.27% reported both tests to be acceptable.

**Conclusions:**

In our adult patients, the new eye-tracking glasses-free display system feasibly screened stereopsis with good repeatability, consistency, and patient acceptance.

## Introduction

Stereopsis is a higher level of binocular function than simultaneous perception and fusion. In addition, it is a rigorous binocular function, as it relies on normal eye alignment and similarly good vision in each eye (1). Anisometropia, ametropia, reduced contrast sensitivity, strabismus, and age may all contribute to stereospsis degeneration (2). Stereoacuity, based on the principle of minimum parallax detected by the eyes, is often used as an index to evaluate stereopsis (3). When testing the threshold of disparity, the two eyes are first separated, which is the basis of the measurement. The common methods for evaluating near stereopsis are Titmus (4) (Stereo Optical Company, Inc., Chicago, Illinois, USA), TNO (5) (Lameris Ootech BV, Ede, Netherlands), and Frisby (6) (Stereotest Ltd., Sheffield, UK). Most of these use printed cards or anaglyphs, and the results are relatively simple, easy to remember, and less reliable (7). In addition, the inherent monocular clues facilitate guessing (8) and increase the false positive rate.

Recently, display technology has developed rapidly (9–11), and some scholars have applied three-dimensional display technology to stereoscopic examination (12–15). Some of these tests still required polarized glasses or shutter goggles and did not overcome limitations such as viewing position restriction, low brightness, and low resolution (9, 14). The glasses-free display system developed by our team, combined with the random-dot and eye-tracking technology, is a new method for near stereoscopic level screening that has not been reported to date.

As a pilot study, we explored the repeatability and consistency of the glasses-free display system applied in myopic adults for stereopsis evaluation without auxiliary glasses, providing a new perspective for clinical stereopsis screening.

## Materials and Methods

### Subjects

A total of 74 patients with myopia, aged 18–44 years, were recruited from the Optometry Department of the Eye Ear Nose and Throat Hospital of Fudan University from November 2020 to March 2021 (male:female, 32:42; best corrected visual acuity, 0 logMAR or better in both eyes). According to the spherical equivalent refraction, patients were divided into two groups: high myopia [HM group; ≤ −6.0 diopters (D)] and moderate-to-low myopia (M-LM group; > −6.0 D). All patients provided written informed consent prior to the study, which was conducted in accordance with the principles of the Helsinki Declaration and approved by the Ethics Committee of the Eye Ear Nose and Throat Hospital of Fudan University.

The inclusion criteria were: (1) 18–45 years of age; (2) best corrected visual acuity of 0 logMAR or better in both eyes; and (3) no strabismus, astigmatism <1.50 D, and anisometropia <2.00 D in both eyes. None of the patients had a history of ocular or systemic disease.

All stereoscopic examinations were performed by the same experienced examiner (XW), and all patients underwent comprehensive ophthalmological examinations in the following order: slit lamp microscopy, best corrected visual acuity, subjective refraction, cover test, three stereotests (glasses-free, polarized, and Titmus test), and dilated fundus examination.

### Examination Procedures

**Figure 1:**
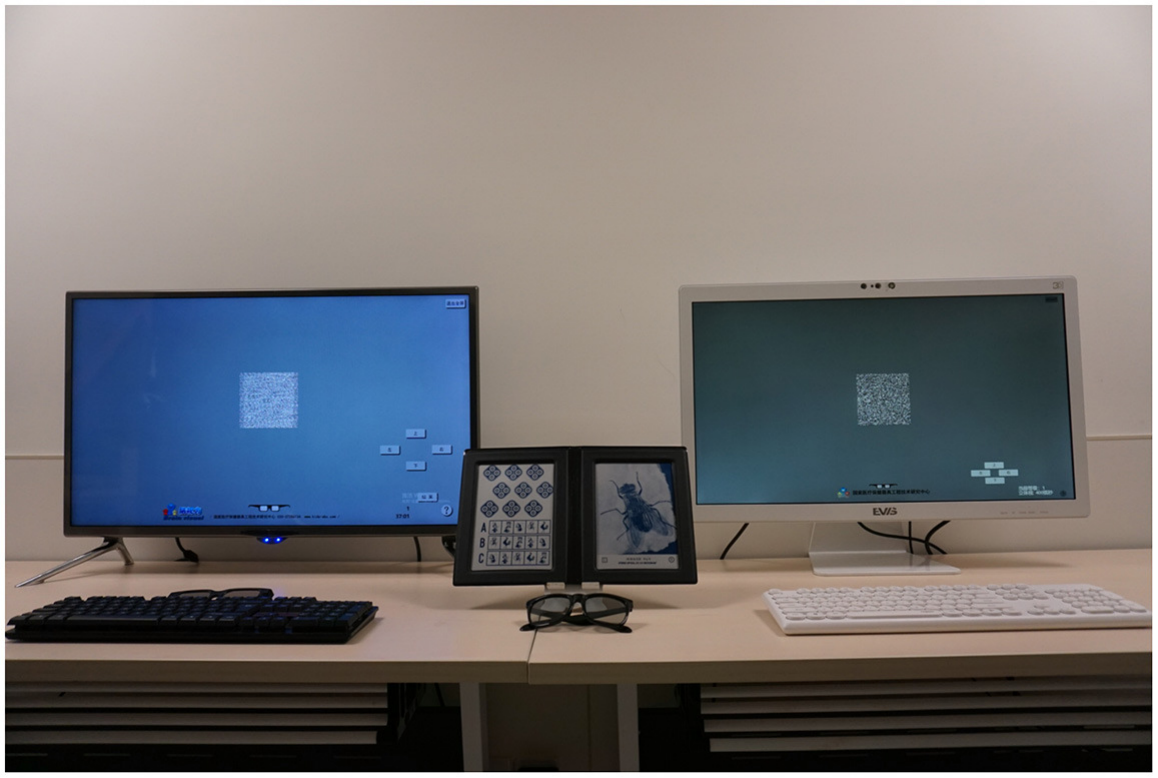
Photographs of glasses-free (right), polarized (left), and Titmus (middle) stereotest systems.

Seventy-four patients underwent glasses-free, polarized, and Titmus stereotests in the same room (Figure 1). The random-dot stimulus target was displayed by the glasses-free and polarization display systems. The three tests were conducted in random order. Measurements for glasses-free, polarized test were taken with interludes of 3 min, and the interval between the two methods was 5 min. The minimum parallax level that patients could distinguish was recorded as the stereoscopic level. The results of the three methods were compared. All patients completed a visual fatigue questionnaire immediately after glasses-free and polarized stereotests to evaluate their visual fatigue level.

### Glasses-Free Stereotest Monitor

**Figure 2:**
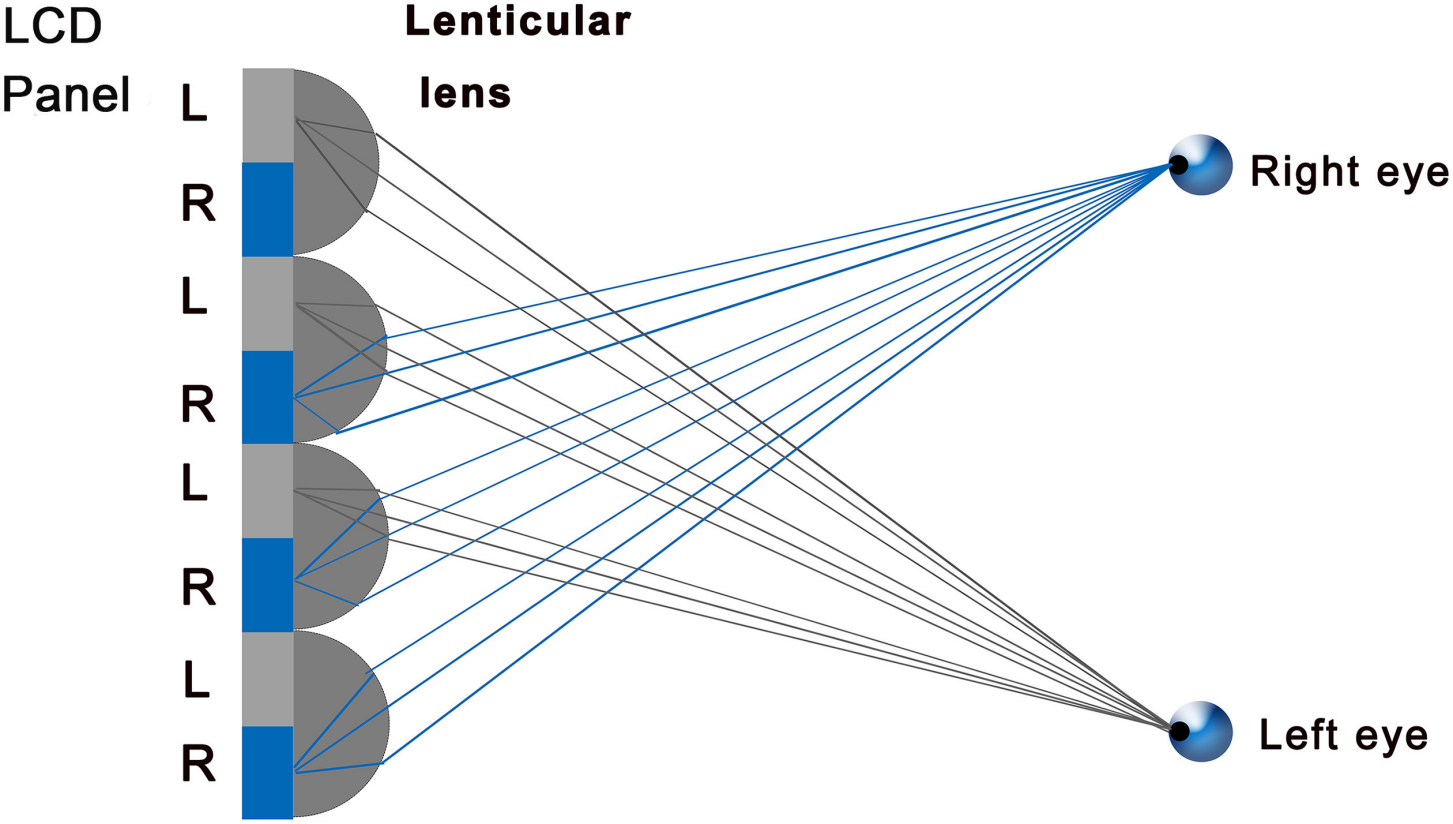
Schematic diagram of image separation by the lenticular lens technique. The gray portion was seen by the left eye; the blue portion was seen by the right eye.

The device consisted of a depth camera and a 28-inch glasses-free display (M090L028; EVIS Co., Inc., Shanghai, China) with a background luminance of 300 cd/m^2^, resolution of 3,840 × 2,160 pixels, and contrast ratio of 1,000:1. The depth camera could acquire the orientation of the eyeballs by eye-tracking technology, including the horizontal, vertical, and relative position of the eyeballs in space. Through the mapping relationship between the position of the eyeballs and the grating parameters, the display then separated the images to the left and right eyes by refraction technology (lenticular lens). This method uses the refraction of light to achieve image division, that is, different pixels/sub-pixels of light to guide the different directions of space (16), resulting in the separation of the left and right images, so that images with different parallax produce stereopsis (Figure 2). During the test, parallax is recorded in pixels and converted into arcsecs using the following formula:


$$\text{Disparity }=\;\frac{\text{arctan }(\frac{\text{n}*\text{w}}{\text{D}})}{\text{π}}{\;}^{*}180^{*}3600\;\text{arcsec}$$

where n is the “E” target offsets between the left and right eye (in pixels), w is the physical width of a pixel on the display, and D is the viewing distance between the patient's eyes and display.

**Figure 3:**
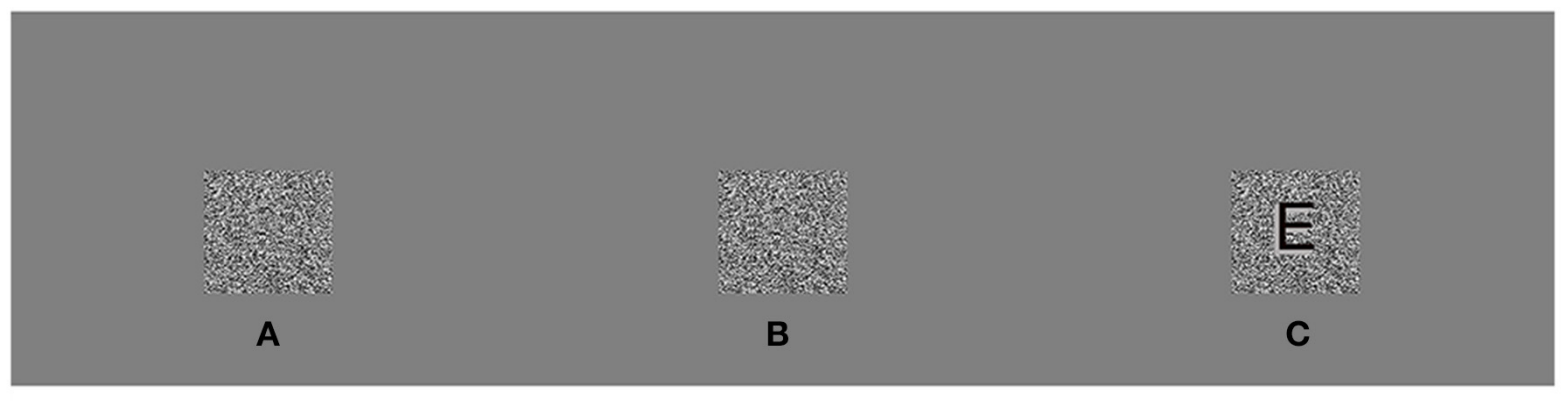
Legend of random-dot stimulus. In the correct test position, the left eye perceived an A and the right eye perceived a B. When the eyes properly fused **(A,B)**, the central optotype “E” seemed to jump out of the plane [**(C)**, “E” was only outlined for visibility and not truly present].

The parameter 3,600 converts from degrees to arcsecs and 180/π converts from radians to degrees. In the glasses-free stereotest monitor, w was 0.16 mm and D was 965 mm. A pixel disparity at 965 mm is about 34′′ (arcsec). Nevertheless, the stereopsis threshold might not be precise enough to test the stereoacuity, however, it can be used as a screening tool, such as Lang stereotest I (550") and II (200", Lang-Stereotest AG, Kusnacht, Switzerland). MATLAB (MathWorks, Co., Inc., Natick, Massachusetts, USA) was used to generate random-dot stimulus targets (Figure 3 consisting of gray background (54 cd/m^2^ average light source) and a central optotype “E” (3° × 3°). Then, four levels of stereopsis were set for this study: 100", 200", 300", and 400".

The patients sat 965 mm away from the screen without wearing auxiliary polarized or red-green glasses, and the device allowed a viewing distance of 600–1,300 mm and a horizontal range of motion of 42°. The stimulus targets were located in the center of the screen, aligned horizontally and vertically with the patients' eyes. If the “E” target was discerned after turning on the eye-tracking program, it meant that the patient's eye was successfully separated and tracked correctly. There were four alternative forced choice tasks for direction options, including up, down, left, and right. Patients pointed out the direction of “E” and pressed the keyboard's direction key corresponding to the position. Testing was conducted according to the order of parallax from large to small. Each level provided four-alternative forced choice tasks, and the identification of three consecutive targets represented a passed test. If any errors occurred, the test ended and the minimum disparity recognizable was recorded.

### Polarized Stereotest Monitor

The “E” target was displayed on a 23-inch polarized monitor (LG2342p; LG Co., Inc., Seoul, Korea) with a resolution of 1,920 × 1,080 pixels and a refresh frequency of 120 Hz. The physical width of a pixel on the display is 0.2652 mm. As the examination distance is changed, the disparity is affected. Therefore, the examination distance was set at 800 mm, so that the “E” target parallax in the polarized stereotest was consistent with that of the glasses-free stereotest. After the patients donned polarized glasses, the examiner covered the patients' left and right eyes separately and questioned them regarding the displayed images to ensure the eyes were properly divided. The target stimulation, testing protocol, and disparity gradient were consistent with those of the glasses-free display stereotest.

### Titmus Stereotest

Fly, animal, and graded circles were applied for quantitative evaluation in the Titmus stereotest. All were administered in an order from fly to graded circles. Polarized glasses were required during evaluation. The detailed processes were consistent with those of a previous report (4).

### Visual Fatigue Questionnaire

The visual fatigue questionnaire was designed and optimized by Chen et al. (17) based on individuals watching a traditional video, facilitating the evaluation of the influence of different displays on the visual fatigue level. The questionnaire includes symptoms of headache, lacrimation, stinging, blurriness, double vision, nausea, grittiness, dizziness, eyestrain, difficulty focusing, and vomiting. Each symptom is scored as follows: none (0), mild (1), less moderate (2), moderate (3), less severe (4), and severe (5). According to the severity of symptoms, visual fatigue is classified into six levels (0–5). To this, we added three open-ended questions: (1) Did you have any other discomfort during the test? (2) Which instrument is more comfortable for you? (3) Which instrument do you prefer? The answers and details for each question were recorded.

### Statistical Analysis

Continuous numerical data are expressed as mean ± standard deviation. To eliminate possible bias due to different parallax gradients and to render them more comparable, stereoacuity results were recorded as one of four levels, including ≤ 100" (level 1), ≤ 200" (level 2), <400" (level 3), and ≥400"/nill (level 4). The Wilcoxon matched pairs test was used to compare the levels of stereopsis between each pair of groups (glasses-free and polarized or Titmus tests). Cohen's Kappa test was used to analyze the agreement between the three groups. Kendall's W test was adopted to evaluate the repeatability of the glasses-free display test in the three consecutive tests. The results for the HM group and the M-LM group were analyzed using the Cochran-Mantel-Haenszel (CMH) chi-square test. SPSS 22.0 Software (SPSS Inc., Chicago, Illinois, USA) was used for analysis. A *P*-value of <0.05 was considered statistically significant.

## Results

The baseline characteristics of the patients are shown in Table 1. There was no significant difference between HM group and M-LM group in sex, age and visual fatigue scores (all *P* > 0.05).

**Table 1:** Baseline patient characteristics.

**Characteristic**	**All patients (*n* = 74)**	**Moderate to low myopia (*n* = 41)**	**High myopia (*n* = 33)**	***P-*value**
**Sex**				0.28
Female	42	21	21	
Male	32	20	12	
Mean age (y)	28.18 ± 6.13	27.90 ± 6.35	28.51 ± 5.93	0.67
**Mean SE (D)**
Right eye	−5.58 ± 2.31	−4.01 ± 1.42	−7.52 ± 1.63	< .001^*^
Left eye	−5.23 ± 2.76	−3.55 ± 1.80	−7.31 ± 2.30	< .001^*^
**Median visual fatigue scores**				0.72
Glasses-free	1 (0, 2.25)	0 (0, 2)	1 (0, 3)	0.107
Polarization	1 (0, 2)	1 (0, 2)	1 (0, 2)	0.111

*y, years old; SE, spherical equivalent; D, diopter; 3-D, 3-dimensional*;

* *P <0.05, P-value, the comparison of high myopia and moderate to low myopia; Mean ± standard deviation; The number of fatigue level in each group was from 0 to 5 scale*.

Comparisons between the glasses-free display and the Titmus, polarized stereotests are shown in Table 2. In terms of glasses-free display, polarized display, and Titmus, the percentages of level 1 were 91, 92, and 85%; the percentages of level 2 were 0, 0, and 8%; the percentages of level 3 were 1, 0, and 0%; and the percentages of level 4 were 8, 8, and 7%, respectively. In the analysis of different levels of stereopsis, there were no significant differences between the glasses-free and polarized, Titmus tests (all *P* > 0.05). The kappa value of 0.494 between the glasses-free 3-D stereotest and the Titmus test indicates moderate agreement (Kappa = 0.493, *P* < 0.001, Table 3). The data obtained from the glasses-free stereotest were in good agreement with that of the polarized stereotest (Kappa = 0.910, *P* < 0.001, Table 3). The test-retest results were identical in 98.6 and 100% of the results were within one stereopsis level, showing good repeatability in the three consecutive stereoacuity tests (*W* = 0.968, *P* < 0.001).

**Table 2:** Comparison of stereopsis level between groups.

	**Glasses-free Stereotest (arcsec)**			
	**≥400"/nill**	** <400"**	**≤200"**	**≤100"**
**Titmus stereotest (arcsec)**
≥400"/nil	4^a^	1^b^	0	0
<400"	0	0	0	0
≤ 200"	0	0	0	6^b^
≤ 100"	1	0	0	62^a^
**Polarized stereotest (arcsec)**
≥400"/nil	5^a^	1^b^	0	0
<400"	0	0	0	0
≤ 200"	0	0	0	0
≤ 100"	0	0	0	68^a^

*3-D, 3-dimensional; The number of patients in each category is embedded in each category*.

a *Identical results on the glasses-free and polarized or Titmus stereotest*.

b *Results within one disparity level on the glasses-free and polarized or Titmus stereotest*.

**Table 3:** Comparative results between groups.

**Comparison stereotest**	**Glasses-free stereotest**			
	**Wilcoxon signed ranks test**		**Cohen's Kappa test**	
	**Z-value**	***P-*value**	**Kappa**	**95% CI**
Polarized stereotest	−1.000	0.317	0.910	0.734–1.000
Titmus stereotest	−1.508	0.132	0.493	0.138–0.753

*CI, confidence interval*.

**Figure 4:**
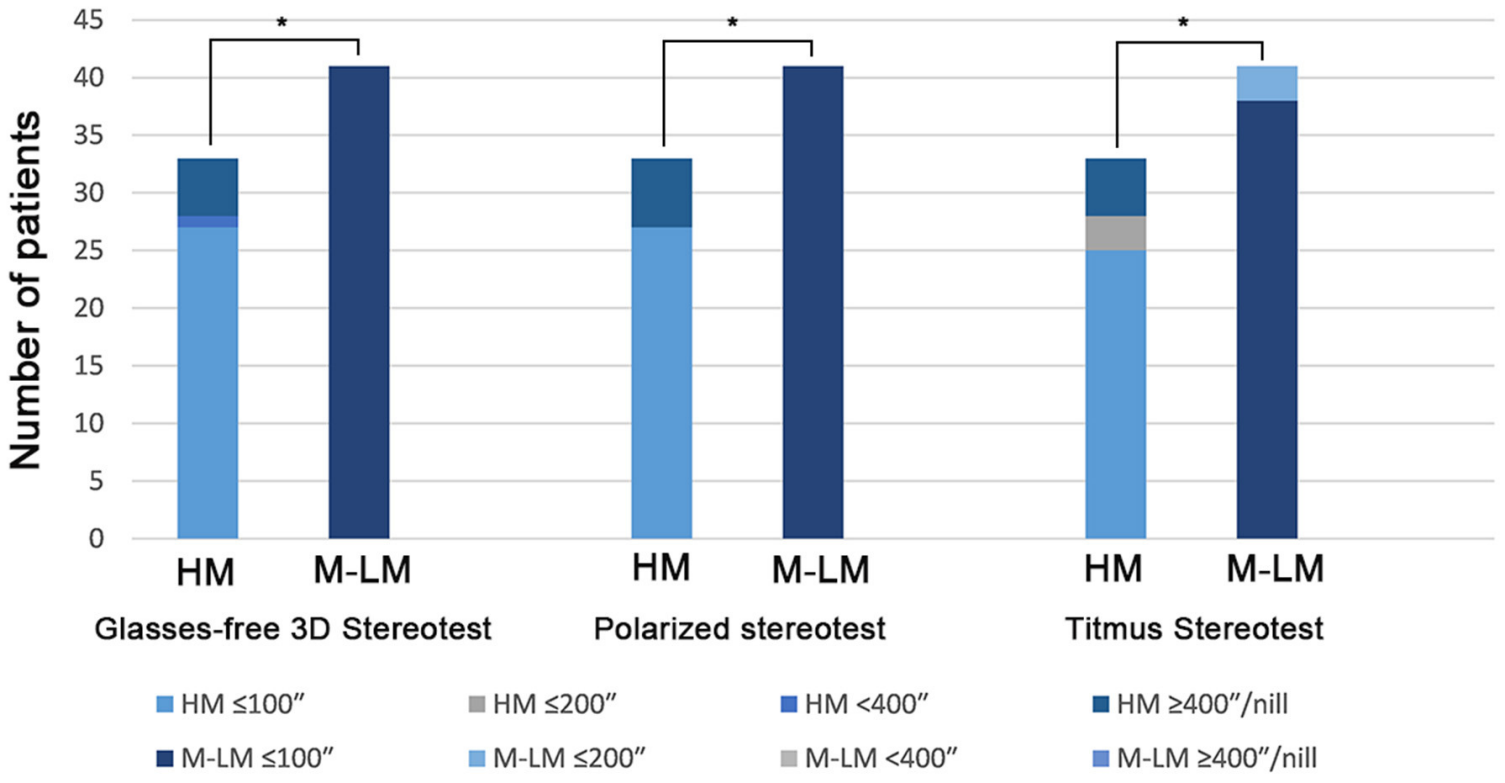
Stereoscopic levels of HM and M-LM groups in three stereotests. HM, high myopia; M-LM, moderate-to-low myopia. *A significant difference between groups.

The differences in stereopsis levels between the HM and M-LM groups are shown in Figure 4. Stereopsis levels of the HM group were significantly poorer than those of the M-LM group in all three tests (all *P* < 0.05).

According to the subjective questionnaire, there was no significant difference in the visual fatigue levels between the glasses-free and polarized tests in all patients (*P* = 0.72). None of the patients had any other discomfort during the test. In addition, compared with the polarized test, 56.76% of patients preferred the glasses-free display and 20.27% reported that both tests were comfortable, with no substantive difference.

## Discussion

Horizontal disparity in binocular retinae determines depth perception, known as stereopsis, which allows accurate judgment of distances. When stereopsis is impaired, the ability to obtain information from the environment is reduced, and this may limit career choices (18). At present, there are many methods to measure stereopsis, and different methods lead to different results, even in the same test (19, 20). Hence, the discussion of a novel stereotest method and apparatus might provide a new perspective for testing. This study was the first to investigate the feasibility and repeatability of a new stereopsis screening method using a glasses-free display combined with random-dot stimulus and eye-tracking technology, which can measure stereopsis in a more natural environment without additional frame restrictions.

Results using the glasses-free display showed a concordance with those of the Titmus and polarized stereotests. Kim et al. (21) used a polarized display to measure distance stereopsis, and the results showed good consistency with those of the Distance Randot Stereotest. Ma et al. (22) measured distance stereoscopic vision using an automated computerized test, showing its consistency with the distance Randot Stereotest. Han et al. (23) utilized a polarization display for near stereotests with results consistent with those of the Titmus test. The above studies were based on contour-based patterns, which might have monocular cues. In that case, the limitations of traditional stereotest methods have not been overcome. Moreover, this new glasses-free display stereotest could measure stereopsis level in a more natural environment and has more location options (60–130 cm viewing distance and 42° horizontal range of motion range) without monocular cues.

Test-retest results for three successive stereotests using the glasses-free display showed high repeatability, indicating that the results were relatively stable. Hess et al. (24) performed a random-dot Mac iPod stereotest, which showed that test-retest results were strongly correlated with little bias. The test-retest results reported by Kim et al. (21) and Ma et al. (22) also supported good repeatability of stereotests. Moreover, the above researches were conducted with the assistance of special glasses and traditional display. Special glasses might not only narrow the visual field, but they also increased extra frame restriction, which could affect the results in children. Meanwhile, the requirement that the patient remained stationary during testing increasing the possibility of result bias. The eye-tracking technology used in this study could alleviate the limitation of the testing position. It could actively follow the position of the eyes and always projected the visual area on the viewer, increasing stability and decreasing the interference from different angles and head movements (horizontal viewing angle of 42°). In addition, the repeatability of results benefited from the lenticular lens technology, which was a type of mature display, and its principle was optically similar to the parallax barrier (25). Because the lenticular lens was transparent, its optical efficiency was much higher than that of the parallax barrier, providing superior brightness (300 cd/m^2^).

In this study, we found that patients with high myopia needed more image parallax information to generate depth perception than patients with moderate-to-low myopia. This was consistent with previous studies (26, 27). Jabbarvand et al. (26) showed that patients with high myopia had poorer baseline stereoacuity scores than patients with low myopia and hyperopia, and the improvement in stereoacuity was most significant in patients with high myopia after photorefractive keratectomy. Guo et al. (27) found that poorer stereoacuity was significantly associated with higher diopter in a study of stereopsis in children in Shandong, China. All patients in our study were adults, who may be more cooperative and reliable than children. In addition, the difference in stereopsis levels between groups might further support the reliability of the glasses-free display. However, the exact mechanism of poor stereopsis in patients with high myopia is unclear and needs further study (28).

Furthermore, there was no statistical difference in visual fatigue levels between the glasses-free and polarized displays. The reason might be that the testing time was short, resulting in inapparent visual fatigue. Compared with the polarized test, a total of 56.76% of patients felt more comfortable with the glasses-free display test and preferred it. Generally, the main cause of visual fatigue from three-dimensional (3D) displays was the accommodation-convergence conflict, which could be ameliorated by increasing the amount of light from different angles simultaneously receiving through the patient's pupil, known as super-multiview technology (29). The glasses-free display combines super-multiview optical separation technology with eye tracking to achieve a high-definition display and to alleviate the above conflicts. Some scholars have proposed that 3D display technology was prone to causing visual fatigue, dry eyes, and other functional eye diseases (30–32). However, few scholars researched visual fatigue when evaluating stereopsis using 3D display technology. The glasses-free display in this study was the first of its kind to be reported, which warranted further research.

There were some limitations in this study. Firstly, this near stereopsis screening only enrolled in adults, and the feasibility for distance stereopsis and for children was unclear. Hence, further research of these issues is warranted. Secondly, as a pilot study of the stereopsis screening, the set threshold may only play a role in a coarse screening, and more accurate disparity must be further studied. Thirdly, the Titmus stereotest could not test 300", it could decrease the test thresholds value from 4 to 3. Nevertheless, only one patient had a stereopsis level of 300" in this study, it will not influence the statistical results. Further study should be conducted to compare with other methods like TNO stereotest. Finally, the sample size of present study was relatively small, larger sample size was needed for further study.

## Conclusion

The new glasses-free display system feasibly screened adult stereopsis with good repeatability, consistency, and comfort, providing more choices for clinical stereotests.

## Data Availability Statement

The original contributions presented in the study are included in the article/supplementary material, further inquiries can be directed to the corresponding author.

## Ethics Statement

All patients provided written informed consent prior to the study, which was conducted in accordance with the principles of the Helsinki Declaration and approved by the Ethics Committee of the Eye, Ear, Nose, and Throat Hospital of Fudan University. The patients/participants provided their written informed consent to participate in this study.

## Author Contributions

FL, JZ, and XZ: conceptualization and critical revision of the manuscript. FL, JZ, TH, and YS: methodology. FL, JZ, ML, JL, and DY: material preparation, data collection, and analysis. LY and YF: software and validation. FL and JZ: writing the manuscript. XZ: funding acquisition and supervision. All authors contributed to the article and approved the submitted version.

## Funding

This study was supported by the National Natural Science Foundation of China (Grant No. 81770955), Joint Research Project of New Frontier Technology in Municipal Hospitals (SHDC12018103), Project of Shanghai Science and Technology (Grant No. 20410710100), Clinical Research Plan of SHDC (SHDC2020CR1043B), Project of Shanghai Xuhui District Science and Technology (2020-015), and Shanghai Engineering Research Center of Laser and Autostereoscopic 3D for Vision Care (20DZ2255000).

## Conflict of Interest

YF was employed by the Shanghai EVIS Technology Co., Ltd., China. The remaining authors declare that the research was conducted in the absence of any commercial or financial relationships that could be construed as a potential conflict of interest. The authors declare that this study received funding from the National Natural Science Foundation of China (Grant No. 81770955), Joint Research Project of New Frontier Technology in Municipal Hospitals (SHDC12018103), Project of Shanghai Science and Technology (Grant No. 20410710100), Clinical Research Plan of SHDC (SHDC2020CR1043B), Project of Shanghai Xuhui District Science and Technology (2020-015), and Shanghai Engineering Research Center of Laser and Autostereoscopic 3D for Vision Care (20DZ2255000). The funder was not involved in the study design, collection, analysis, interpretation of data, the writing of this article or the decision to submit it for publication.

## Publisher's Note

All claims expressed in this article are solely those of the authors and do not necessarily represent those of their affiliated organizations, or those of the publisher, the editors and the reviewers. Any product that may be evaluated in this article, or claim that may be made by its manufacturer, is not guaranteed or endorsed by the publisher.
